# pH-regulated nuclear F-actin assembly during ferroptosis

**DOI:** 10.3389/fcell.2025.1699698

**Published:** 2025-12-10

**Authors:** Menghao Qiao, Zhipeng Yan, Jiewei Huang, Yu Fang, Kun Xu, Lingbo Cao, Haiying Mai, Na Li, Yanmei Li, Yunmiao Guo, Junqi Huang

**Affiliations:** 1 Key Laboratory of Regenerative Medicine of Ministry of Education, Institute of Aging and Regenerative Medicine, Department of Developmental and Regenerative Medicine, College of Life Science and Technology, Jinan University, Guangzhou, China; 2 Zhanjiang Institute of Clinical Medicine, Central People’s Hospital of Zhanjiang, Guangdong Medical University Zhanjiang Central Hospital, Zhanjiang, China

**Keywords:** ferroptosis, cytoskeleton, actin, nuclear actin, pH

## Abstract

**Introduction:**

Ferroptosis is an iron-dependent form of programmed cell death driven by lipid peroxidation and loss of membrane integrity, frequently modeled with small molecules such as RSL3 that inhibit Glutathione Peroxidase 4 (GPX4). Filamentous actin (F-actin) exists within the nucleus, modulating transcription, nuclear mechanics, and chromatin organization, yet its behavior during ferroptosis remain unexplored.

**Methods:**

Here, we show that nuclear F-actin assembles in HT-1080 cells undergoing RSL3-induced ferroptosis, visualized by phalloidin, SiR-actin, anti-Actin staining, and live 3D/time-lapse imaging of a nuclear actin chromobody (nAC-TagGFP2).

**Results:**

Mechanistically, nuclear G-actin increased during ferroptosis, and Importin-9 (IPO9) knockdown markedly reduced nuclear F-actin, indicating an import-dependent mechanism. Concurrently, cytoplasmic F-actin underwent substantial remodeling. Overexpression of a polymerization-defective cytoplasmic β-actin mutant (R62D) slightly delayed ferroptosis, whereas nuclear-targeted mutants had no effect, suggesting nuclear F-actin is a concomitant, not causative, feature. Notably, extracellular NaHCO_3_ or NaOH suppressed nuclear F-actin formation, while a pH-sensitive reporter revealed progressive intracellular acidification during ferroptosis, favoring nuclear F-actin assembly.

**Discussion:**

These findings reveal nuclear F-actin assembly driven by cytoplasmic actin remodeling, nuclear import, and intracellular acidification, uncovering a previously unrecognized feature of ferroptotic cell death.

## Introduction

In recent years, ferroptosis has emerged as an important form of programmed cell death (Dixon et al., 2012; Calhoon et al., 2025; Cañeque et al., 2025; Tonnus et al., 2025; Wang et al., 2025; Yang et al., 2025; Zhou et al., 2025). Ferroptosis is implicated in various pathological conditions, including tumor progression, ischemia-reperfusion injury, neurodegenerative diseases, and skin disorders (Stockwell et al., 2017). In Parkinson’s disease, for example, excessive iron accumulation and lipid peroxidation in the substantia nigra are associated with dopaminergic neuronal loss (Chen et al., 2019). In cancer, ferroptosis exhibits a dual role, some tumor cells resist ferroptosis via antioxidant responses (e.g., upregulation of NRF2 and SLC7A11), while therapeutic induction of ferroptosis offers a promising strategy to overcome treatment resistance (Zhang et al., 2022; Gu et al., 2024). Unlike classical modes of cell death such as apoptosis, necroptosis, or pyroptosis, ferroptosis is characterized by lipid peroxidation, along with plasma membrane rupture (Stockwell et al., 2017). Mechanistically, increased intracellular ferrous iron (Fe^2+^) promotes lipid peroxidation through Fenton chemistry, while inhibition of GPX4 or inactivation of the cystine/glutamate antiporter system Xc^−^ are critical triggers of ferroptosis (Yang et al., 2014). These events disrupt intracellular redox homeostasis and activate downstream cell death pathways. Numerous regulators modulate cellular sensitivity to ferroptosis. For example, dysregulation of ion-handling proteins such as transferrin receptor and ferritin can affect ferroptosis progression (Gao et al., 2015). Additionally, p53 promotes ferroptosis by repressing SLC7A11 transcription, while NRF2 counteracts ferroptosis by inducing antioxidant gene expression (Jiang et al., 2015; Sun et al., 2016; Zhang, 2025). Despite growing interest, the full mechanistic landscape of ferroptosis remains incompletely understood (Stockwell, 2022).

Actin, one of the most abundant and evolutionarily conserved cytoskeletal proteins in eukaryotic cells, plays a central role in maintaining cell shape, motility, endocytosis, and signal transduction (Dominguez and Holmes, 2011). Notably, the actin cytoskeleton has been implicated in programmed cell death regulation (Ren et al., 2021). For instance, phosphorylation of heat shock protein β1 (HSPB1 or HSP27) promotes actin polymerization and cytoskeletal reorganization, which in turn limits iron uptake and confers resistance to erastin-induced ferroptosis (Huot et al., 1996; Sun et al., 2015). The nuclear translocation of NRF2 is facilitated by the cytoplasmic F-actin-Keap1 axis (Fan et al., 2017; van der Kammen et al., 2017). Signaling pathways implicated in regulating ferroptosis, such as EGFR and MAPK, are also closely linked with the actin cytoskeleton (Tomas et al., 2006; Poursaitidis et al., 2017).

Although traditionally nuclear actin was considered to exist mainly in monomeric (G-actin) form and primarily regulate transcription, recent advances revealed that nuclear F-actin also plays essential roles. For example, nuclear F-actin have been observed during DNA damage repair, where they facilitate the mobilization of damaged DNA loci to more favorable repair environments (Caridi et al., 2018; Hurst et al., 2019). Furthermore, nuclear F-actin also contributes to chromatin decondensation, nuclear architecture remodeling, transcriptional activation via RNA polymerase II clustering, post-mitotic chromatin reorganization, T cell activation, decidualization in endometrial stromal cells, and embryonic development (Sen et al., 2024; Ulferts et al., 2021; Wang et al., 2019; Kyheröinen and Vartiainen, 2020).

In this study, we employed ferroptosis-sensitive HT-1080 fibrosarcoma cells to investigate the spatiotemporal dynamics of nuclear F-actin during ferroptosis. Using a nuclear actin chromobody-based imaging system, we report that ferroptosis induced by RSL3 triggers nuclear F-actin bundle assembly. This nuclear F-actin assembly is regulated by the bicarbonate content of the culture medium and the extracellular/intracellular pH, unveiling a previously unrecognized link between ferroptosis, nuclear actin cytoskeletal remodeling, and pH homeostasis.

## Materials and methods

### Cell culture

HT-1080 human fibrosarcoma cells were obtained from the Shanghai Institute of Cell Biology, Chinese Academy of Sciences (Shanghai, China). Cells were maintained in complete growth medium consisting of Dulbecco’s Modified Eagle Medium (DMEM, high glucose, Gibco) supplemented with 10% (v/v) fetal bovine serum (FBS, ExCell Bio) and 1% (v/v) penicillin-streptomycin solution (100 U/mL penicillin and 100 μg/mL streptomycin, Beyotime). The medium was stored at 4 °C and pre-warmed to 37 °C before use. Cells were cultured in 25 cm^2^ or 75 cm^2^ sterile tissue culture flasks (NEST) and incubated at 37 °C in a humidified atmosphere containing 5% CO_2_ and 95% air. For Passaging, when cells reached 80%–90% confluency, remove medium, rinse once with PBS, add 0.25% Trypsin-EDTA (500 μL per 25 cm^2^ flask), incubate at 37 °C for 30 s until cells round up, stop reaction with 3 mL complete medium, gently pipette to single-cell suspension, centrifuge at 1,000 rpm for 5 min, discard supernatant, resuspend in fresh medium and reseed at 1:3–1:6 split ratio. For cryopreservation: harvest log-phase cells as above, centrifuge, discard supernatant, resuspend in 90% FBS and 10% DMSO at 1-2 × 10^6^ cells per 1 mL, aliquot into cryovials, transfer to −80 °C overnight, then plunge into liquid nitrogen.

### Chemicals and reagents

RSL3 (Selleck, 0.5–2 μM), ferrostatin-1 (Fer-1, Selleck, 2–5 μM), and deferoxamine (DFO, Selleck, 100 μM) were dissolved in DMSO and used at indicated concentrations. Other reagents included SiR-actin (Cytoskeleton), Alexa Fluor 488–DNase I (Thermo), and Hoechst 33342 (Beyotime). Stock solutions were prepared at appropriate concentrations, aliquoted into centrifuge tubes, sealed, protected from light and stored at −80 °C until use.

### Generation of stable cell lines

HT-1080 cells were transduced with lentiviruses encoding nuclear actin chromobody (TagGFP; ChromoTek), β-actin-NLS-GFP (wild-type and mutants: R62D, S14C), or mOrange2-pH reporter constructs. Plasmids were cloned into pcDNA3.1, pCMV or pMXs vectors. For lentivirus production, 293T cells were co-transfected with the plasmid and packaging plasmids (psPAX2 and pMD2.G) using a standard calcium phosphate or PEI-based transfection method. Viral supernatants were collected at 48 and 72 h post-transfection, filtered through a 0.45 μm PVDF membrane, and concentrated using 50 mg/mL PEG8000 (Amresco). HT-1080 cells were infected with viral supernatant and selected with puromycin (1 μg/mL; Thermo Fisher) to establish stable lines.

### F-actin, G-actin, and anti-Actin staining

Cells were seeded on 4-well confocal chambers at 5 × 10^4^ cells/well and allowed to adhere overnight. For F-actin detection, cells were fixed with pre-warmed 4% paraformaldehyde at 37 °C for 15 min, gently aspirated, rinsed 3 times with PBS to remove residual aldehyde, incubated with Alexa Fluor 647- or Rhodamine-labeled phalloidin (1:200 in 1% BSA-PBS) for 30 min at room temperature in the dark, and finally washed 3 times with PBS. For G-actin staining, fixed cells were permeabilized with 0.5% Triton X-100 in PBS for 10 min at room temperature, rinsed 3 times with PBS, incubated with Alexa Fluor 488–DNase I (1:300, Cytoskeleton) for 45 min at room temperature in the dark to label G-actin, and gently washed 3 times with PBS. For anti-Actin antibody staining, fixed cells were permeabilized with 0.5% Triton X-100 in PBS for 10 min, washed 3 times with PBS, blocked in 5% BSA for 1 h, and incubated overnight at 4 °C with primary anti-Actin antibody (1:500), followed by Alexa Fluor-conjugated secondary antibodies (1:2000; Invitrogen) at room temperature in the dark for 1 h. Cells were then washed 3 × 5 min with PBS. Nuclei were finally stained with 2 μg/mL Hoechst 33342 for 10 min at room temperature, gently washed with PBS, and kept in a small residual volume of PBS to prevent drying.

### Cell death assay

Cell death was assessed by staining with SYTOX Green and Hoechst 33342 followed by calculation of the ratio of SYTOX Green-positive (dead cells) to total (Hoechst-positive) nuclei. Briefly, cells were seeded in 96-well plates and allowed to adhere for 24 h, then treated with the appropriate compounds. After the designated treatment period, cells were co-stained with SYTOX Green (5 μg/mL) and Hoechst 33342 (2 μg/mL) and incubated at room temperature for 20 min. Following staining, images were acquired and cells were counted using Cytation imaging system (Agilent BioTek).

### Construction of actin overexpression and mutant vectors

The pMXs-β-actin-R62D-NLS2-P2A-mCherry-IRES2-BSD lentiviral vector (hereafter referred to as NLS-Actin R62D) was subjected to site-directed mutagenesis (Beyotime) to generate β-actin-WT-NLS2-P2A-mCherry (NLS-Actin WT) and β-actin-S14C-NLS2-P2A-mCherry (NLS-Actin S14C) lentiviral vectors using the following primers:

Sense primer: agagcaagagaggcatcctcaccctgaagta

Anti-sense primer: tacttcagggtgaggatgcctctcttgctct

S14C (Ser→Cys)

Sense primer: tcgacaacggctgcggcatgtgcaa

Anti-sense primer: ttgcacatgccgcagccgttgtcga

To construct a plasmid overexpressing cytoplasmic actin mutant, we performed reverse PCR to remove the tandem NLS sequences.

### siRNA-mediated gene silencing

For siRNA transfection, the siRNA powder was centrifuged at 15,000 rpm for 1 min and dissolved in DEPC water to a concentration of 125 μL per 33 μg. HT-1080 cells were seeded in 6-well plates at a density of 150,000 cells per well and incubated at 37 °C with 5% CO_2_ for 24 h. The transfection mixture was prepared by diluting 7.5 µL Lipofectamine RNAiMAX (Invitrogen) and 1.5 µL siRNA separately in 125 µL Opti-MEM, combining the two solutions (total 250 µL), and incubating the mixture for 20 min at room temperature. The resulting complex was added to the wells, and cells were incubated for 12 h. The medium was then replaced with fresh DMEM for subsequent experiments.

### Live-cell imaging and time-lapse microscopy

Live imaging was performed using an LSM 880 (Zeiss) or Olympus FV3000 confocal microscope equipped with environmental control (37 °C, 5% CO_2_). Objectives included 20× air and 60× oil immersion. Z-stacks were acquired with a step size of 0.5–2 μm. Time-lapse videos were taken with imaging intervals of 5–20 min, as indicated. For pH monitoring, HT-1080 cells expressing mOrange2 were imaged in different buffer systems with adjusted pH.

### Intracellular pH measurement

The mOrange2 fluorescent plasmid (a pH-sensitive variant derived from *Discosoma* sp. red fluorescent protein) was packaged into lentiviral particles and transduced into HT-1080 cells. Stable cell lines were established by selection with puromycin. HT-1080 cells expressing the mOrange2 fluorescent tag were evenly seeded into 6- or 12-well plates and cultured for 24 h. The culture medium was then replaced with medium containing the indicated drugs, and cells were incubated at 37 °C in a humidified incubator with 5% CO_2_ for the specified duration. After treatment, cells were washed with PBS and collected into centrifuge tubes. Collected samples were analyzed by flow cytometry using 561 nm excitation; fluorescence was recorded with detectors FL2 (585 nm, PE channel) and FL3 (670 nm, PE-Texas Red channel). A minimum of 10,000 cells per well were acquired for each analysis.

### Image processing and quantification

All images were processed and analyzed using Fiji/ImageJ. Nuclear F-actin dynamics were visualized by maximum-intensity projection unless otherwise specified. Three-dimensional datasets were analyzed and rendered with Imaris.

### Statistical analysis

GraphPad Prism (v9.0) was used for all statistical analyses. Data are presented as mean ± SEM. Unpaired two-tailed Student’s t-tests were used for comparisons between two groups. For multiple comparisons, one-way or two-way ANOVA was performed. Significance levels are indicated on each graph. All experiments were conducted with 3 biological replicates.

## Results

### Nuclear F-actin is assembled during RSL3-induced ferroptosis

**FIGURE 1:**
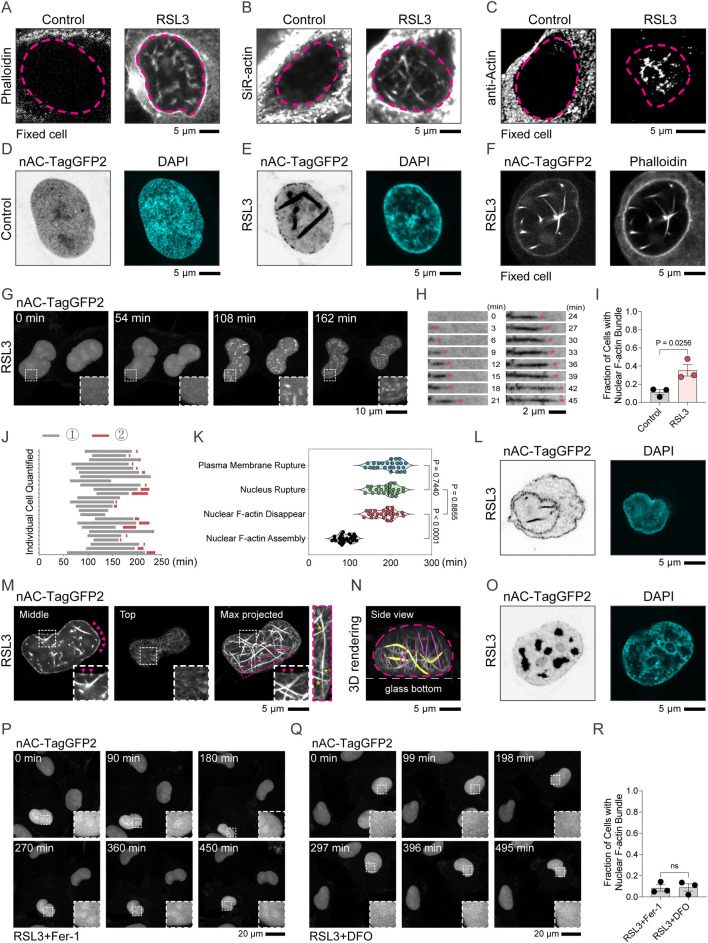
Nuclear F-actin assembly is induced during ferroptosis. **(A)** HT-1080 cells treated with 1 μM RSL3 for 4 h were subjected to Phalloidin staining, and nuclear F-actin was imaged under high exposure conditions using a confocal microscope. The nucleus of a representative cell in each condition is delineated by a magenta dashed line. **(B)** HT-1080 cells treated with 1 μM RSL3 for 4 h were subjected to SiR-actin staining, and nuclear F-actin was imaged using a confocal microscope. The nucleus of a representative cell in each condition is delineated by a magenta dashed line. **(C)** HT-1080 cells treated with 1 μM RSL3 for 4 h were subjected to immunostaining with anti-Actin antibodies, and nuclear F-actin was imaged using a confocal microscope. The nucleus of a representative cell in each condition is delineated by a magenta dashed line. **(D,E)** Representative focused microscopy images of HT-1080 cells stably expressing nuclear actin chromosome-TagGFP2 (nAC-TagGFP2) after treatment with 2 μM DMSO or 2 μM RSL3 for 2.5 h. **(F)** Representative confocal microscopy images of phalloidin staining and nAC-TagGFP2 co-localization in HT-1080 cells overexpressing nAC-TagGFP2 after treatment with 2 μM RSL3 for 2.5 h. **(G,H)** Confocal time-lapse series shows dynamic nuclear F-actin assembly during ferroptosis. The nAC-TagGFP2 was treated with 2 μM RSL3. **(I)** Percentage of cells with nuclear F-actin from **(G)**. **(J)** Time quantification of 25 cells upon 2 μM RSL3 treatment. Gray indicates the time between nuclear F-actin assembly and disappear indicated by nAC. Magenta nuclear membrane rupture, and plasma membrane rupture. **(K)** Time quantification of nuclear F-actin assembly, disappear, nuclear membrane rupture, and plasma membrane rupture. **(L)** Hoechst-stained HT-1080 cells stably expressing nAC-TagGFP2 and treated with 2 μM RSL3 for 4 h were subjected to nuclear F-actin imaging under high-exposure conditions using a confocal microscope. **(M)** HT-1080 cells stably expressing nAC-TagGFP and treated with 2 μM RSL3 for 2.5 h were subjected to 3D confocal reconstruction analysis. Lower panels display magnified views of regions demarcated by white rectangles. Red triangles indicate F-actin near the nuclear periphery. Right panels display magnified views of regions demarcated by red rectangles. **(N)** Imaris 3D reconstructions show orientation-specific nuclear F-actin organization. **(O)** High-exposure confocal microscopy of Hoechst-stained HT-1080 cells expressing nAC-TagGFP2 after 2 μM RSL3 treatment for 2.5 h **(P,Q)** Confocal microscopy images of HT-1080 cells stably expressing nAC-TagGFP following co-treatment with 2 μM RSL3 and either 2 μM Ferrostatin-1 or 100 μM DFO. **(R)** Quantification of fluorescence intensity in HT-1080 cells from **(P,Q)**.

We first investigated whether nuclear F-actin forms during RSL3-induced ferroptosis in HT-1080 cells. Phalloidin staining, a canonical method for detecting F-actin, predominantly labels cytoplasmic actin filaments, which can obscure weaker nuclear F-actin signals. Nonetheless, under high camera exposure conditions, we were able to detect nuclear F-actin structures in RSL3-treated cells (Figure 1A). To further confirm this, we performed live-cell staining with SiR-actin (Figure 1B), another widely used F-actin probe, or used anti-Actin antibodies (Figure 1C). Consistently, nuclear F-actin structures were also observed in ferroptotic cells using these approaches. To specifically trace nuclear actin, we generated a stable HT-1080 cell line expressing the nuclear actin chromobody linked with TagGFP2 (nAC-TagGFP2). Under basal conditions, nAC-TagGFP2 signals exhibited partial localization in the nucleolus, possibly reflecting endogenous G-actin localization, but were otherwise diffusely distributed throughout the nucleoplasm, suggesting the absence of prominent nuclear F-actin bundles in HT-1080 cells (Figure 1D). After exposure to RSL3, nuclear F-actin structures assembled in ferroptotic nuclei (Figure 1E), with the nAC-TagGFP2 signal colocalizing with phalloidin staining (Figure 1F). Further time-lapse imaging revealed robust formation of nuclear F-actin bundles upon RSL3 induction (Figures 1G,H). Quantification showed that ∼35% of HT-1080 cells assembled nuclear F-actin bundles in DMEM supplemented with serum upon ferroptosis induction (Figure 1I). Ferroptotic nuclear F-actin appeared ∼90 min after RSL3 treatment, persisted for an additional ∼90 min, and disassembled concomitantly with nuclear and plasma membrane rupture (Figures 1J,K). Upon nuclear rupture, nAC-TagGFP2 was released into the cytoplasm, where it partially labeled cytoplasmic F-actin (Figure 1L).The prolonged existence of these ferroptotic nuclear F-actin is intriguingly much longer than the transient nuclear F-actin reported in previously published literature (Baarlink et al., 2013; 2017; Wang et al., 2019). Further 3D confocal reconstruction analysis demonstrated that these ferroptotic nuclear F-actin bundles preferentially formed near the ventral side of the nucleus and displayed differential thickness depending on subnuclear localization, thinner F-actin near the nuclear periphery and thicker bundles within the nucleoplasm (Figure 1M). These filaments occasionally crossed each other and displayed both horizontal and vertical orientations relative to the ventral side of cells (Figures 1M, N). Notably, some ferroptotic nAC-TagGFP2 signals also formed clump-like structures within the nucleoplasm regions, where they excluded DAPI staining, suggesting potential chromatin exclusion or redistribution (Figure 1O). Treatment with ferroptosis inhibitors ferrostatin-1 (Fer-1) or deferoxamine (DFO) markedly inhibited nuclear F-actin assembly, indicating that this phenotype is tightly associated with ferroptotic induction (Figures 1P–R). Together, our results showed the presence of nuclear F-actin bundles during the progression of RSL3-induced ferroptosis.

### Nuclear F-actin assembly does not affect ferroptosis sensitivity

**FIGURE 2:**
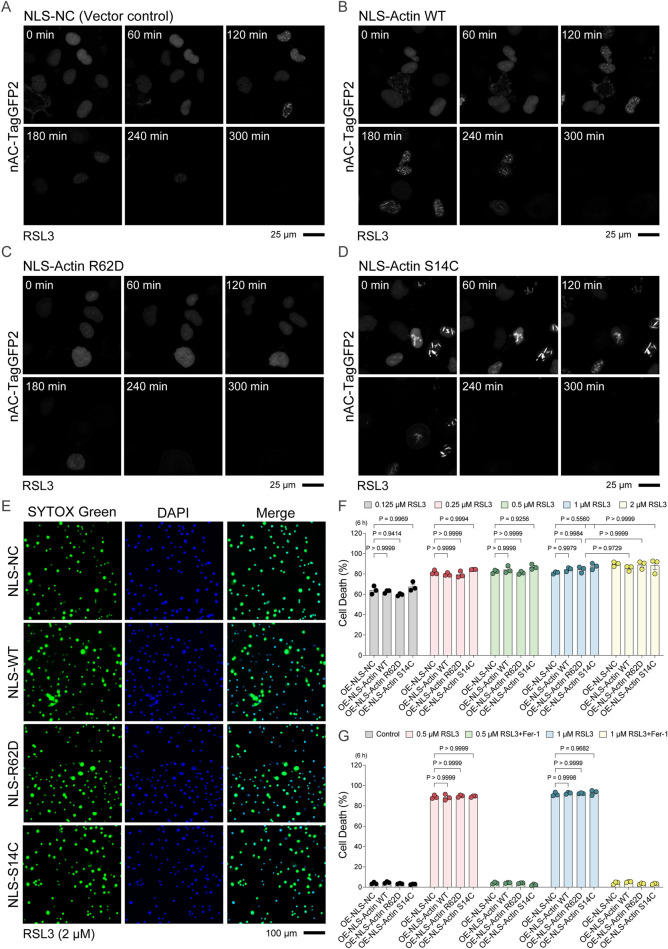
Stabilization or disruption of nuclear F-actin does not affect ferroptotic sensitivity. **(A–D)** In HT-1080 cells overexpressing NLS-actin variants with the indicated point mutations, time-lapse imaging was performed using the GFP channel of a confocal microscope following 2 μM RSL3 treatment. **(E)** Representative Hoechst and SYTOX Green staining wide-field images of HT-1080 cells overexpressing NLS-actin variants with the indicated point mutations, treated with 2 μM RSL3 for 6 h. **(F)** Cell death in HT-1080 cells overexpressing NLS-actin variants with the indicated point mutations, treated with a concentration gradient of 0.125/0.25/0.5/1/2 μM RSL3 for 6 h. **(G)** Cell death in HT-1080 cells overexpressing NLS-actin variants with the indicated point mutations, treated with RSL3 at concentrations of 0.5 or 1 μM, either alone or in combination with 2 μM Ferrostatin-1, for 6 h.

To determine whether nuclear F-actin formation influences ferroptotic cell fate, we overexpressed actin mutants in HT-1080 cells via adenoviral transduction. NLS-Actin WT, the polymerization-defective mutant NLS-Actin R62D, and the filament-stabilizing mutant NLS-Actin S14C were used, along with an NLS-negative control (empty vector) (Figures 2A–D). Time-lapse confocal imaging of cells co-expressing nAC-TagGFP2 revealed that RSL3-induced nuclear F-actin assembly was reduced in cells overexpressing NLS-Actin R62D (Figure 2C). In contrast, wild-type (WT)- and S14C-overexpressing cells assembled nuclear F-actin bundles during ferroptosis (Figures 2B,D). Interestingly, under unstimulated conditions, S14C-overexpressing cells occasionally exhibited much thicker nuclear F-actin bundles, consistent with its known stabilizing effect (Figure 2D). To examine whether these nuclear actin mutants influence ferroptotic cell fate, HT-1080 cells overexpressing the mutants (without nAC-TagGFP2) were stained with SYTOX Green and Hoechst, and the SYTOX Green/Hoechst ratio was used to quantify cell death (Figures 2E–G). However, this co-staining assay revealed no significant differences in RSL3-induced cell death rates between the control, WT, R62D, and S14C groups (Figures 2F,G), suggesting that nuclear F-actin assembly could be a concurrent feature rather than a determinant of ferroptotic sensitivity.

### Ferroptotic nuclear F-actin assembly arises from cytoplasmic F-actin disassembly and G-actin nuclear import

**FIGURE 3:**
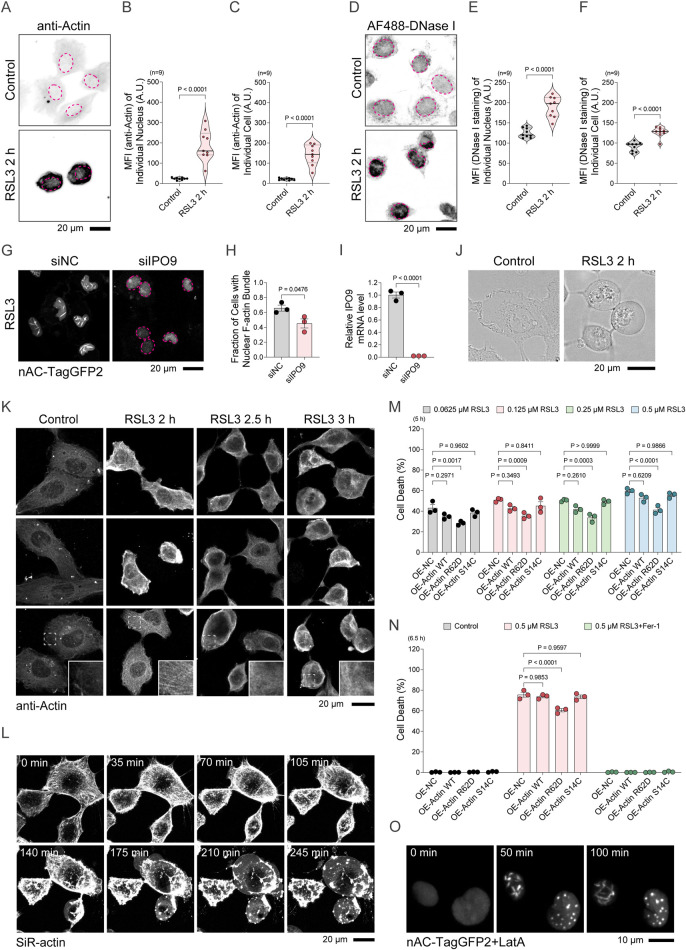
Nuclear F-actin formation is driven by cytoplasmic actin remodeling and G-actin nuclear import. **(A)** HT-1080 cells treated with 2 μM DMSO or 2 μM RSL3 for 2 h were subjected to immunostaining with anti-Actin antibodies, and nuclear F-actin was imaged using a confocal microscope. The nucleus of a representative cell in each condition is delineated by a magenta dashed line. **(B,C)** Quantification of the nuclear actin and cellular actin concentration detected by the anti-Actin antibody from **(A)**. **(D–F)** Alexa Fluor 488–DNase I staining detects nuclear G-actin and total cellular G-actin amount, and fluorescence intensity was quantified using image analysis software. The nucleus of a representative cell in each condition is delineated by a magenta dashed line. **(G)** Confocal imaging of nuclear F-actin in HT-1080 nAC-TagGFP2 cells after siRNA-mediated IPO9 knockdown or siNC control, followed by 2 μM RSL3 treatment for 2.5 h. **(H)** Percentage of cells with nuclear F-actin from **(G)**. **(I)** Relative IPO9 mRNA levels in cells transfected with control siRNA (siNC) or IPO9 siRNA (siIPO9) for 48 h, measured by qPCR. **(J)** Brightfield imaging of cellular morphological alterations following DMSO or 2 μM RSL3 treatment for 2 h. **(K)** Confocal SUM-projected images of anti-Actin antibody staining in HT-1080 cells treated with 2 μM RSL3 for 2, 2.5, and 3 h. **(L)** Time-lapse confocal imaging of SiR-actin staining in HT-1080 cells treated with 2 μM RSL3. **(M)** Cell death in HT-1080 cells overexpressing actin variants with the indicated point mutations, treated with a concentration gradient of 0.0625/0.125/0.25/0.5 μM RSL3 for 5 h. **(N)** Cell death in HT-1080 cells overexpressing actin variants with the indicated point mutations, treated with 0.5 μM RSL3 or in combination with 2 μM Ferrostatin-1 for 6.5 h. **(O)** Confocal imaging of HT-1080 cells overexpressing nAC-TagGFP2 after co-treatment with 2 μM latrunculin A.

To explore the mechanism underlying nuclear F-actin assembly during ferroptosis, we first assessed nuclear actin concentration using anti-Actin antibody. RSL3-treated HT-1080 cells exhibited a marked increase in nuclear actin levels, as well as in the total cellular actin pool (Figures 3A–C). Similarly, staining with the G-actin specific dye, Alexa Fluor 488-conjugated deoxyribonuclease I (AF488-DNase I), suggested an increase of nuclear G-actin level and the whole cell G-actin level (Figures 3D–F). Given that cytoplasmic G-actin can be actively transported into the nucleus, the rise of ferroptotic nuclear G-actin may suggest an enhanced nuclear import of G-actin from the cytoplasm. Cytoplasmic G-actin import into the nucleus requires specialized transport factors, such as IPO9. Active maintenance of nuclear actin by IPO9 supports transcription (Dopie et al., 2011). To test whether IPO9 is involved in ferroptosis-associated nuclear F-actin assembly, we knocked down IPO9 expression and observed a marked reduction in nuclear F-actin assembly during RSL3-induced ferroptosis (Figures 3G–I). These results support a role for IPO9-mediated G-actin nuclear import in driving ferroptotic nuclear F-actin assembly. Since ferroptosis triggers dramatic changes in cell morphology (Figure 3J), we hypothesized that cytoplasmic F-actin disassembly enhances G-actin nuclear import and fuels nuclear F-actin assembly. Using anti-Actin antibody staining at different timepoints, we examined cytoplasmic F-actin alterations in RSL3-treated cells (Figure 3K). Early in ferroptosis, transient increases in cytoplasmic F-actin intensity were observed. However, this was followed by progressive F-actin disassembly at later stages. Time-lapse imaging with SiR-actin further confirmed this cytoplasmic F-actin remodeling pattern during RSL3-induced ferroptosis (Figure 3L). To determine whether cytoplasmic F-actin dynamics influence ferroptosis sensitivity, we overexpressed cytoplasmic actin mutants in HT-1080 cells. Compared to wild-type actin, the polymerization-deficient R62D mutant partially delayed RSL3-induced ferroptosis, whereas the F-actin-stabilizing S14C mutant had no significant effect (Figures 3M,N). Consistent with this, both prior studies and our own findings in HT-1080 cells showed that treatment with latrunculin A (LatA), an actin-depolymerizing agent that induces cytoplasmic F-actin disassembly, led to a concurrent increase in nuclear F-actin assembly, suggesting a tightly coupled inverse relationship between cytoplasmic and nuclear F-actin dynamics (Figure 3O) (Hurst et al., 2024). Together, these results suggest that cytoplasmic actin cytoskeleton remodeling contributes to nuclear F-actin assembly during ferroptosis.

### pH-regulated ferroptotic nuclear F-actin assembly

**FIGURE 4:**
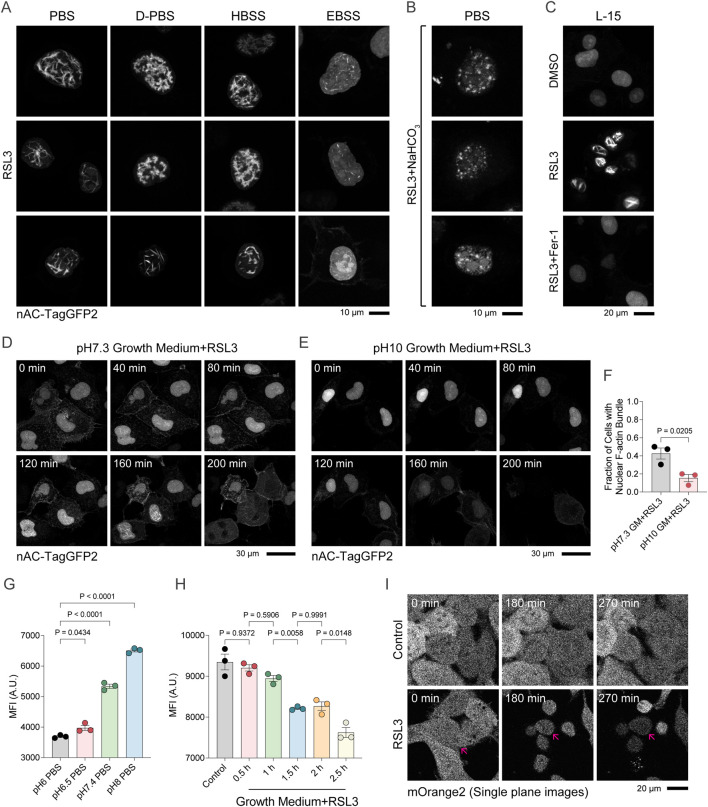
Extracellular bicarbonate and alkaline pH suppress ferroptosis-associated nuclear F-actin formation. **(A)** Confocal imaging of HT-1080 cells overexpressing nAC-TagGFP2 following 2 μM RSL3 treatment under different buffering solutions for 3 h. **(B)** Confocal imaging of HT-1080 cells overexpressing nAC-TagGFP2 treated with 2 μM RSL3 in PBS supplemented with NaHCO_3_ for 3 h. **(C)** Confocal imaging of HT-1080 cells overexpressing nAC-TagGFP2 following treatment with 2 μM RSL3, 2 μM DMSO, or 2 μM RSL3 plus 2 μM Ferrostatin-1 (Fer-1) in bicarbonate-free L-15 medium for 3 h. **(D,E)** Confocal imaging of HT-1080 cells overexpressing nAC-TagGFP2 treated with 2 μM RSL3 in GM(DMEM + FBS) adjusted to pH 7.3 and pH 10. **(F)** Percentage of cells with nuclear F-actin from **(D,E)**. **(G)** Detection of fluorescence intensity in HT-1080 cells overexpressing the pH-sensitive fluorescent reporter mOrange2 across a PBS-adjusted pH gradient. **(H, I)** Confocal imaging and fluorescence quantification analysis in HT-1080 cells overexpressing mOrange2 following treatment with 2 μM DMSO or 2 μM RSL3. Arrows indicate the same cell during time-lapse imaging.

We serendipitously observed that the proportion of cells exhibiting nuclear F-actin assembly was markedly higher in phosphate-buffered saline (PBS) than in standard growth medium (DMEM supplemented with fetal bovine serum). This prompted us to systematically compare nuclear F-actin assembly across various buffered solutions (Figure 4A). Among these, Earle’s Balanced Salt Solution (EBSS) supported the least nuclear F-actin assembly during RSL3-induced ferroptosis, whereas PBS, Dulbecco’s Phosphate-Buffered Saline (D-PBS), and Hanks’ Balanced Salt Solution (HBSS) supported more prominent nuclear F-actin assembly. A key compositional difference is that EBSS contains a much higher concentration of sodium bicarbonate (NaHCO_3_) than the other buffers tested (Table 1). Moreover, we found that with decreasing NaHCO_3_ concentrations (EBSS, 26 mM > HBSS, 4.2 mM > D-PBS = PBS = 0 mM), nuclear F-actin structures display a mixture of long, straight bundled filaments alongside diffuse, mesh-like, and clump-like aggregates (Figure 4A). Supplementation of NaHCO_3_ into PBS abolished the ferroptotic long nuclear F-actin bundles (Figure 4B). Conversely, robust bundled nuclear F-actin was again observed in bicarbonate-free L-15 medium following RSL3 treatment (Figure 4C). Given the central role of NaHCO_3_ in regulating pH, we next adjusted the extracellular pH using NaOH instead of NaHCO_3_ to isolate the effect of alkalinity. Increasing extracellular pH significantly suppressed nuclear F-actin assembly in ferroptotic cells (Figures 4D–F), suggesting that pH, rather than bicarbonate itself, regulates this process. Because extracellular pH directly influences intracellular pH, we hypothesized that nuclear F-actin assembly correlates with intracellular acidification. Using HT-1080 cells stably expressing the pH-sensitive fluorescent protein mOrange2 (Sarker et al., 2022), we found that intracellular pH in HT-1080 cells closely tracked changes in extracellular pH (Figure 4G). Importantly, progressive intracellular acidification was observed during RSL3-induced ferroptosis (Figures 4H,I), which may create a favorable environment for nuclear F-actin assembly. Together, these findings demonstrate that ferroptosis-associated nuclear F-actin assembly is regulated by pH and may be favored by intracellular acidification.

**TABLE 1:** Composition of various buffer solutions (PBS, D-PBS, HBSS, EBSS).

Concentration (mM)	PBS	D-PBS	HBSS	EBSS
NaCl	136.000	137.931	137.931	116.359
Na_2_HPO_4_	8.000	8.059	0.338	0.000
NaH_2_PO_4_	0.000	0.000	0.000	1.017
KCl	2.600	0.000	5.333	5.366
D- glucose	0.000	0.000	5.555	5.551
NaHCO_3_	0.000	0.000	4.166	26.187
KH_2_PO_4_	2.000	1.470	0.441	0.000

## Discussion

Actin is one of the most evolutionarily conserved and abundant proteins in eukaryotic cells. It dynamically switches between monomeric and filamentous states to form bundles and complex networks, which are precisely regulated in space and time to orchestrate key biological processes such as cell motility, cell division, intracellular transport, signal transduction, and programmed cell death (Gibieža and Petrikaitė, 2021). While the functions of cytoplasmic F-actin have been extensively characterized, nuclear F-actin has historically been less understood, with its roles only recently gaining broader recognition.

As our understanding of the cytoskeleton and cell death pathways deepens, growing evidence suggests that dynamic remodeling of the actin cytoskeleton plays integral roles in the regulation of cell death programs (Ren et al., 2021). For instance, during apoptosis, actin and actin-binding proteins participate in DNA fragmentation, apoptotic body formation, and activation of caspase cascades (King and Campellone, 2023). Ferroptosis has also been linked to cytoskeletal components in prior studies, such as actin-mediated regulation of intracellular iron levels (Luscieti et al., 2017).

Our study reveals the formation of nuclear F-actin bundles as a prominent feature of RSL3-induced ferroptosis. However, functional assays using actin mutants indicate that nuclear F-actin assembly is not essential for ferroptotic sensitivity. Instead, this process appears to be a consequence of ferroptotic signaling, driven by cytoplasmic F-actin disassembly, increase of nuclear G-actin level, and intracellular acidification.

The observation that intracellular acidification promotes nuclear F-actin assembly aligns with prior studies showing that actin polymerization is pH-sensitive (Wioland et al., 2018; Wang et al., 1989). Our work expands on this concept by demonstrating that extracellular pH and bicarbonate buffering systems, commonly used in cell culture, can significantly influence nuclear F-actin assembly. This may partially explain why nuclear F-actin structures are often underrepresented under standard bicarbonate-rich culture conditions. Importantly, our findings thus suggest that pH and buffer composition must be carefully considered when studying nuclear F-actin biology.

Mechanistically, ferroptosis triggers extensive remodeling of the cytoplasmic actin cytoskeleton, including transient assembly followed by large-scale disassembly. This cytoskeletal collapse may release G-actin into the cytoplasm, which can be transported into the nucleus via IPO9. Once in the nucleus, G-actin undergoes polymerization, especially under acidic intracellular conditions. These nuclear F-actin bundles appear to exhibit organized orientation and may influence chromatin organization and nuclear architecture during ferroptosis.

Several important questions remain. First, the specific actin nucleators or formins that drive nuclear F-actin assembly during ferroptosis have yet to be identified. Second, it is unclear whether similar nuclear F-actin structures form in pathological contexts, such as neurodegenerative diseases or tumors undergoing ferroptosis. Third, the functional consequences of nuclear F-actin formation, e.g., whether it affects transcription, chromatin accessibility, or DNA integrity, require further mechanistic investigation. Fourth, whether nuclear F-actin is also present and functional in other forms of regulated cell death, including cuproptosis and triaptosis, remains to be determined (Tsvetkov et al., 2022; Swamynathan et al., 2024; Qiao et al., 2025). Future studies should also explore the roles of different nuclear actin isoforms (e.g., γ-actin) and utilize CRISPR-based knock-in models to precisely examine the effects of actin mutants *in situ*.

In conclusion, we identify nuclear F-actin assembly as a previously unrecognized feature of ferroptosis, regulated by cytoplasmic actin remodeling, intracellular acidification, and the extracellular environment. These findings provide a new perspective on the interplay between actin cytoskeletal dynamics and ferroptotic signaling.

## Data Availability

The original contributions presented in the study are included in the article/supplementary material, further inquiries can be directed to the corresponding authors.
